# Term Delivery of a Complete Molar Pregnancy with a Coexistent Normal Pregnancy

**DOI:** 10.1155/2019/5090565

**Published:** 2019-10-07

**Authors:** Manisha Chhetry, Aruna Pokharel, Amar Nath Chaudhary

**Affiliations:** ^1^Department of Obstetrics and Gynecology, BP Koirala Institute of Health Science, Nepal; ^2^Department of Obstetrics and Gynecology, Nobel Medical College and Teaching Hospital, Nepal

## Abstract

Twin pregnancy with a complete mole and a coexistent normal fetus reaching term is a rare occurrence. We report a case of a 21-yrs G2P1L0 un-booked patient at 39 weeks who was referred for the same condition diagnosed incidentally on ultrasound scan which showed a singleton pregnancy in breech presentation with a normal placenta and a heterogeneous cystic lesion seen anteriorly, suggesting a coexistent molar pregnancy. Cesarean section was done, and a healthy male baby was delivered with a grossly normal placenta and a second placenta with grape like vesicles. Histopathology confirmed the diagnosis of complete mole and normal placenta. Postoperative period was uneventful, and the patient was kept on beta hcg follow-up to monitor progression to gestational trophoblastic neoplasia, but it normalized by 12 weeks.

## 1. Introduction

Twin pregnancy with complete mole and a coexistent normal fetus is a rare entity with incidence quoted between 1/22,000 and 1/100,000 pregnancies [1–7]. These pregnancies rarely reach term and are usually complicated with spontaneous abortions, congenital malformations, preterm labor, early-onset preeclampsia, sudden fetal loss, and risk of progressing to persistent gestational trophoblastic neoplasia to name a few [8–14]. With the recent development of various assessment modalities, complete hydatiform mole with a coexistent live fetus continuation of pregnancy to term and delivery is becoming an acceptable option.

## 2. Case

**Figure 1:**
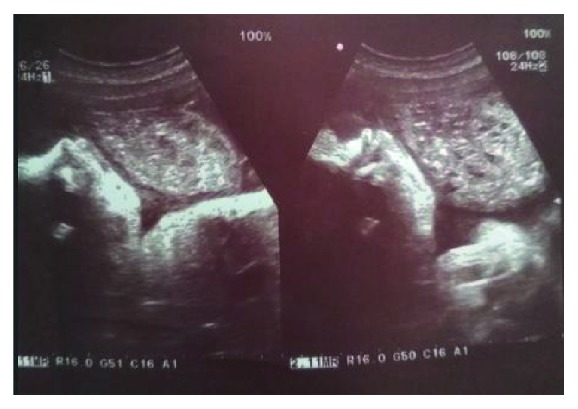
Ultrasound showing a heterogeneous cystic lesion anteriorly and a normal fetus.

**Figure 2:**
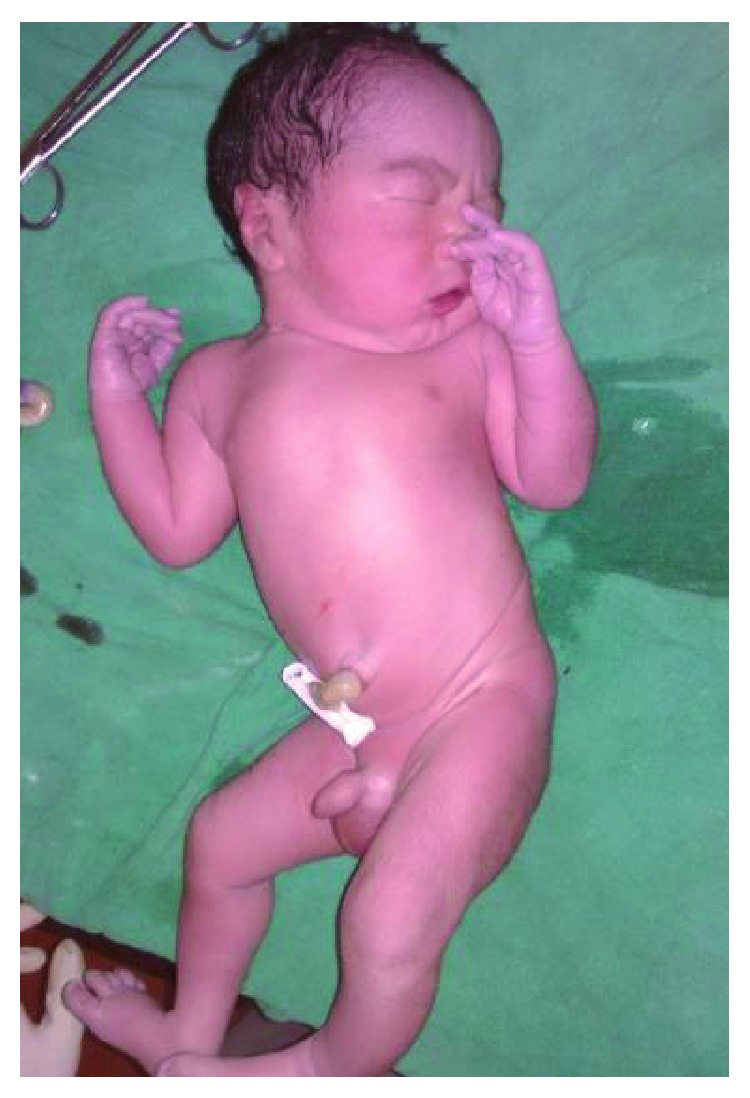
Coexistent healthy baby.

**Figure 3:**
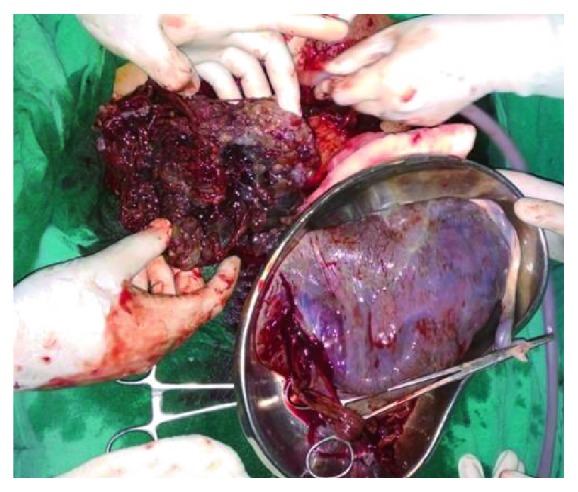
Normal placenta and second sac showing placenta with grape like vesicles and no fetal poles.

A 21-yrs un-booked G2P1L0 patient at 39 weeks POG was referred from a remote hilly district for pregnancy with coexistent molar pregnancy. The patient had one previous term intrauterine fetal death delivered at home 3 yrs back, and even in this pregnancy, she only visited the local health post at term where the condition was suspected on a routine obstetric scan by the attending doctor and she was referred to our center. On examination, she was normotensive; there was no pallor or oedema. Her uterus was term size with breech presentation with reassuring fetal heart rate. Ultrasound showed a singleton pregnancy with fetal biometry corresponding to 36 weeks in breech presentation with adequate amniotic fluid and a normal placenta; additionally, a heterogeneous cystic lesion was seen anteriorly with features, suggesting a coexistent molar pregnancy (Figure 1). Her *β* hcg at admission was 2, 55,000 mIU, thyroid function test was normal (TSH 2 mIU/L), there was no proteinuria, and chest X-ray was normal. The counseling of patient and spouse was done, and she was taken up for cesarean section. She delivered a healthy male baby of 3.6 kg (Figure 2) with a grossly normal placenta, while the second sac had only placental tissue with grapelike vesicles (Figure 3). The intraoperative and postoperative period was uneventful, and the histopathology of placental tissue showed complete mole, while the second placenta was normal. The post-evacuation bhcg dropped to 51,000 mIU/ml. The patient was kept on two-weekly bhcg follow-up as surveillance to progression to persistent gestational trophoblastic neoplasia, but it normalized by 12 weeks.

## 3. Discussion

Twin pregnancy with one healthy fetus and a coexistent molar pregnancy is an uncommon occurrence [1–7]. This condition is not only rare but also complex and challenging to manage as there are no clear consensuses regarding the same. Successful diagnosis requires critical interpretation of clinical symptoms, ultrasound findings, and biochemical and cytogenetic studies [8, 9]. In our patient, the condition was diagnosed in ultrasound scan in the late third trimester. Diagnosis was late as the patient did not visit any health facility for antenatal checkup. Ideally, the patient should have undergone a prenatal diagnosis by chorionic villous sampling in early pregnancy to exclude a case of partial mole and confirm uniparental disomy with cytogenetic studies [10]. This becomes important because it has been proved that complete and partial moles are two distinct pathological entities [11, 12].

All patients diagnosed with this condition have to be counseled, and pregnancy can be continued in those patients who elect to continue under proper supervision. Molar pregnancy with a coexistent live fetus is associated with severe pregnancy complications [8], so antenatal surveillance becomes all the more important, an aspect which was missed in our patient due to her late presentation. A literature review suggests that common complications include vaginal bleeding, preeclampsia, hyperemesis, hyperthyroidism, preterm birth, and fetal loss [8, 13–15]. There is more risk of persistent disease in this group of patients so they need to be followed up even after delivery [13, 15, 16].

Although there are differences in opinion regarding the management of this group of patients due to rarity of cases but it is reasonable to evaluate for metastatic disease, rule out triploidy as these fetuses are at high risk of malformation and closely monitor for possible complications like pre-eclampsia [13, 17]. Mandatory placental examination by skilled pathologist is needed in the index as well as the subsequent deliveries to rule out choriocarcinoma. The standard post-molar bhcg follow-up is to be done. Continuation of pregnancy in patients with complete mole and coexistent normal fetus is an acceptable option, provided proper surveillance is done. Proper counseling of patients and respecting patient's choice also becomes an important aspect of management.
